# Fast acquisition of a polysaccharide fermenting gut microbiome by juvenile green turtles *Chelonia mydas* after settlement in coastal habitats

**DOI:** 10.1186/s40168-018-0454-z

**Published:** 2018-04-10

**Authors:** Patricia Campos, Miriam Guivernau, Francesc X. Prenafeta-Boldú, Luis Cardona

**Affiliations:** 1 IRBio and Department of Evolutionary Biology, Ecology and Environmental Science, Faculty of Biology, University of Barcelona, Avenida Diagonal 643, 08028 Barcelona, Spain; 2 GIRO, Institute of Agrifood Research and Technology (IRTA), Torre Marimon, E-08140 Caldes de Montbui, Barcelona, Spain

**Keywords:** Tetrapods, Herbivorous, Microbial communities, *Chelonia mydas*, 16S rRNA, Fermentation

## Abstract

**Background:**

Tetrapods do not express hydrolases for cellulose and hemicellulose assimilation, and hence, the independent acquisition of herbivory required the establishment of new endosymbiotic relationships between tetrapods and microbes. Green turtles (*Chelonia mydas*) are one of the three groups of marine tetrapods with an herbivorous diet and which acquire it after several years consuming pelagic animals. We characterized the microbiota present in the feces and rectum of 24 young wild and captive green turtles from the coastal waters of Brazil, with curved carapace length ranging from 31.1 to 64.7 cm, to test the hypotheses that (1) the ontogenetic dietary shift after settlement is followed by a gradual change in the composition and diversity of the gut microbiome, (2) differences exist between the composition and diversity of the gut microbiome of green turtles from tropical and subtropical regions, and (3) the consumption of omnivorous diets modifies the gut microbiota of green turtles.

**Results:**

A genomic library of 2,186,596 valid bacterial 16S rRNA reads was obtained and these sequences were grouped into 6321 different operational taxonomic units (at 97% sequence homology cutoff). The results indicated that most of the juvenile green turtles less than 45 cm of curved carapace length exhibited a fecal microbiota co-dominated by representatives of the phyla *Bacteroidetes* and *Firmicutes* and high levels of *Clostridiaceae*, *Prophyromonas*, *Ruminococaceae*, and *Lachnospiraceae* within the latter phylum. Furthermore, this was the only microbiota profile found in wild green turtles > 45 cm CCL and in most of the captive green turtles of any size feeding on a macroalgae/fish mixed diet. Nevertheless, microbial diversity increased with turtle size and was higher in turtles from tropical than from subtropical regions.

**Conclusions:**

These results indicate that juvenile green turtles from the coastal waters of Brazil had the same general microbiota, regardless of body size and origin, and suggest a fast acquisition of a polysaccharide fermenting gut microbiota by juvenile green turtles after settlement into coastal habitats.

**Electronic supplementary material:**

The online version of this article (10.1186/s40168-018-0454-z) contains supplementary material, which is available to authorized users.

## Background

Herbivory has evolved independently in several groups of tetrapods belonging to diverse evolutionary lineages [1]. Unlike some invertebrates, tetrapods do not express hydrolases for cellulose and hemicellulose [2], and hence, the independent acquisition of herbivory required the establishment of new endosymbiotic relationships between tetrapods and microbes [1, 3–5]. As a consequence, the composition, abundance, and diversity of the gut microbiota of herbivorous tetrapods vary widely across groups, reflecting not only their evolutionary relationships but also their foraging habits and the location of the cavity of fermentation into the gut–hindgut vs. foregut fermenters [6–8].

Several groups of tetrapods have recolonised the marine environment after independent evolution in land, but only three of them are herbivores: sirenians (manatees and the dugong), the marine iguana (*Amblyrhynchus cristatus*), and the green turtle (*Chelonia mydas*). Sirenian diet is dominated by seagrasses [9–12] which are vascular plants rich in cellulose [13, 14]. Consequently, sirenians host microorganisms producing the enzymes needed for the fermentative digestion of cellulose [15, 16]. On the other hand, marine iguanas feed only on macroalgae [17]. The cell wall of macroalgae differs from that of seagrasses and other vascular plants in the abundance of sulfated polysaccharides and alginic acid and low levels of cellulose [18]. As a consequence, the microbiota of marine iguanas is characterized by the presence of some specific groups of methanogens and differs largely from that of terrestrial iguanas, despite a close evolutionary relationship [3]. Green turtles exhibit a much larger dietary flexibility than sirenians and marine iguanas, as they undergo a major ontogenetic dietary shift from animal-based to plant-based diets following settlement in coastal areas [19–25]. Nevertheless, they also exhibit a high level of regional variability in the degree of omnivory after settlement and the relative importance of seagrasses and seaweeds in their diets [20, 21, 23, 26–34].

The acquisition of a specialized microbiota is facilitated by lactation and intimate calve/mother relationships in mammals [35] and the consumption of conspecific excrements in marine iguanas [17]. On the contrary, the solitary lives of green turtles may delay the acquisition of a specialized gut microbiota, which in combination with the higher body temperature of larger turtles in winter may explain the improved digestibility and assimilation of plant material as green turtles grow [13, 20]. This is because green turtles are ectothermic, and the body temperature of inactive adult green turtles can be 2 °C above water temperature thanks to gigantothermy [36], whereas that of juveniles matches that of the environment [37]. It has also been suggested that mixed seagrass/macroalgae diets are uncommon in green turtles because the entirely different structure of polysaccharides in their cell walls would require different compositions of the gut microbiota [38]. In such case, frequent and short-term shifts in diet may reduce the efficiency of plant digestion [39].

Unfortunately, very little is known about the gut microbiota of green turtles, how it changes after settlement in coastal areas in association to the increase in the consumption of plant material, and the influence of turtle diet on microbiota composition. The only information available to our knowledge is about the microbiota present in the cloaca of pelagic and recently settled green turtles, which reveals a high prevalence of *Proteobacteria* and a low occurrence of bacteria associated to the fermentation of structural polysaccharides [40]. In this study, we characterize the microbiota present in the feces and rectum of young wild and captive green turtles from Brazil to test the hypotheses that (1) the ontogenetic dietary shift after settlement is followed by a gradual change in the composition and diversity of the gut microbiome, (2) differences exist in the composition and diversity of the gut microbiome of green turtles from tropical and subtropical regions, and (3) the consumption of omnivorous diets modifies the gut microbiota of green turtles.

## Methods

### Study area

Two different areas of Brazil were sampled in February to March 2016. Most samples (*n* = 20) were collected from subtropical Ubatuba (23° 26′ S, 45° 05′ W), in the northern coast of the state of Sao Paulo. Rocky reefs and sandy beaches dominate the coastline of Ubatuba [41]. A few additional samples (*n* = 5) were collected from tropical Praia do Forte (12° 38′ S 38° 05′ W), located 70 km from Salvador do Bahia. The coastline is characterized by the presence of shallow coral reefs with substantial air exposition during low tide [42].

### Sampling

Fecal samples were collected from 8 turtles held in captive at the facilities of Projeto Tamar at Ubatuba and 11 wild turtles from Ubatuba. Some wild green turtles were captured alive in weirs (“*Cercos flutuantes*”) used by local fishermen and consisting on fixed nets attached to the seafloor [43], and others were captured alive through free diving by members of Projeto Tamar (www.tamar.org.br), as part of the long-term study on the abundance and habitat use of green turtles along the Brazilian coast. After capture, curved carapace length (CCL) was measured with a flexible tape (CCL, notch to tip) and turtles were moved to the facilities of Projeto Tamar in Ubatuba. These turtles were confined in individual PVC tanks until the moment they defecated, between 24 and 36 h after capture, and then released back to the sea at the same place of capture. Tanks had been previously disinfected with regular bleach. The core of each fecal pellet was accessed using sterilized forceps and sampled with a swab, to reduce as much as possible contamination from water. Additionally, rectal samples (*n* = 5) were collected with a swab during the necropsy of recently dead turtles at Praia do Forte.

Fecal and rectal samples were stored at 4 °C immediately after collection and then at − 20 °C until DNA extraction. No buffers were used. All procedures were non-invasive and conducted in accordance with guidelines from the Projeto TAMAR and ICMBio.

### DNA extraction and next-generation sequencing

DNA was extracted from a subsample of 0.25 g from each fecal or rectal sample using the PowerSoil DNA kit (MO BIO Laboratories, Carlsbad, CA, USA) following the manufacturer’s instructions. All DNA extracts were kept frozen at − 20 °C until further analysis. Massive bar-coded 16S rRNA gene-based libraries in the *Eubacteria* domain were sequenced by using the MiSeq Illumina platform (Molecular Research DNA LP, Shallowater, USA). These gene libraries were constructed by targeting the V1–V3 hypervariable regions with the primer set 27F (5′-AGRGTTTGATCMTGGCTCAG-3′)/519R (5′-GTNTTACNGCGGCKGCTG-3′) as previously described in [44]. The obtained DNA reads were compiled in FASTq files for further bioinformatic processing. Trimming of the 16S rRNA barcoded sequences into libraries was carried out using QIIME software version 1.8.0 [45]. Quality filtering of the reads was performed at Q25, the default set in QIIME, prior to the grouping into operational taxonomic units (OTU) at a 97% sequence homology cutoff. The following steps were performed using QIIME: Denoising of sequence data using Denoiser [46], picking up of OTU reference sequences via the first method of the UCLUST algorithm [47] and, for sequence alignment and chimera detection, processing by PyNAST [48] and ChimeraSlayer [49]. OTUs were then taxonomically classified using BLASTn against GreenGenes and RDP (Bayesian Classifier) databases and compiled into each taxonomic level [50].

### Biostatistical methods

A general lineal model (GLM) using locality (Ubatuba vs. Praia do Forte) as a fixed factor and turtle curved carapace length as a covariable was used to test the hypothesis that the microbial diversity of wild green turtles increases with turtle size and varies across localities. A general lineal model using origin (captive vs. wild) as a fixed factor and turtles curved carapace length as a covariable was used to test the hypothesis that the microbial diversity of green turtles increases with turtle size and differs between captive and wild green turtles from subtropical Ubatuba. GLMs were run in IBM SPSS Statistics 23. Multivariate principal coordinate analysis (PCoA) based on Bray-Curtis similarity distances was carried out on the OTUs incidence matrix using the CANOCO software package, version 5 (Microcomputer Power, Ithaca, NY, USA), to identify clusters of green turtles differing in the community structure of their microbiomes.

## Results

The gut microbiome of 24 green turtles ranging in curved carapace length (CCL) from 31.1 to 64.7 cm was studied. A genomic library of 2,187,066 valid eubacterial 16S rRNA reads was obtained from their feces (Additional file 1). These sequences were grouped into 6321 different OTUs (at 97% sequence homology cutoff), ranging from 473 to 1952 in individual turtles (Table 1). The Good’s coverage estimator on the percentage of the total species (as OTUs) represented in any given sample was above 98%, indicating that the observed species encompassed a very significant proportion of the entire sample populations. With this respect, the number of expected OTUs (Chao 1) ranged from 959 to 2818 and the Shannon index from 2.17 to 5.38 (Table 1). The number of recovered and expected OTUs in wild turtles form Praia del Forte was larger than those in wild turtles from Ubatuba and increased significantly with curved carapace length in both areas according to GLM (Table 2). However, the indices of microbial diversity did not differ between wild and captive turtles from Ubatuba (GLM; OTUs: F_2,18_ = 1.750, *p* = 0.205; Chao1: F_2,18_ = 1.922, *p* = 0.179; Shannon: F_2,18_ = 2.445, *p* = 0.118).

**Table 1:** Descriptors of bacterial diversity in fecal and rectal samples of juvenile green turtles *Chelonia mydas* from Brazil

Study area	Origin	Turtle	CCL (cm)	Total reads	OTUs	Coverage (%)	Shannon (ave ± SD)^a^	Chao1 (ave ± SD)^a^
Praia do Forte–BA	Wild	PF1	31.1	70,792	1589	99	4.69 ± 0.006	2015 ± 78
Praia do Forte–BA	Wild	PF2	35.0	111,405	1794	99	4.17 ± 0.008	1790 ± 88
Praia do Forte–BA	Wild	PF3	38.8	70,850	1997	98	5.14 ± 0.006	2523 ± 85
Praia do Forte–BA	Wild	PF4	40.0	90,045	1911	99	4.22 ± 0.008	2148 ± 87
Praia do Forte–BA	Wild	PF5	44.0	89,351	2211	98	5.05 ± 0.007	2466 ± 90
Ubatuba–SP	Wild	UB6	37.0	127,862	1217	99	2.16 ± 0.009	1071 ± 69
Ubatuba–SP	Wild	UB7	39.7	68,389	601	99	2.59 ± 0.006	956 ± 80
Ubatuba–SP	Wild	UB8	40.0	98,513	1954	99	4.70 ± 0.007	2053 ± 77
Ubatuba–SP	Wild	UB9	41.3	76,055	1947	99	4.82 ± 0.006	2389 ± 90
Ubatuba–SP	Wild	UB10	44.7	61,852	598	99	3.08 ± 0.005	953 ± 67
Ubatuba–SP	Wild	UB11	45.0	119,273	2150	99	4.37 ± 0.008	2036 ± 93
Ubatuba–SP	Wild	UB12	47.0	119,764	2206	99	4.47 ± 0.008	2050 ± 81
Ubatuba–SP	Wild	UB13	53.3	84,889	2006	99	4.60 ± 0.008	2264 ± 79
Ubatuba–SP	Wild	UB14	54.2	107,097	1670	99	3.24 ± 0.009	1657 ± 71
Ubatuba–SP	Wild	UB15	58.3	90,582	1951	99	4.53 ± 0.007	2187 ± 90
Ubatuba–SP	Wild	UB16	61.4	79,361	2179	98	5.15 ± 0.006	2540 ± 83
Ubatuba–SP	Captivity	UB17	32.5	103,168	2284	99	4.55 ± 0.008	2355 ± 88
Ubatuba–SP	Captivity	UB18	34.9	121,100	1481	99	2.88 ± 0.009	1374 ± 75
Ubatuba–SP	Captivity	UB19	38.6	56,987	1723	99	4.85 ± 0.005	2447 ± 77
Ubatuba–SP	Captivity	UB20	40.0	123,937	1442	99	2.79 ± 0.009	1302 ± 71
Ubatuba–SP	Captivity	UB21	41.3	101,478	2436	98	4.68 ± 0.008	2549 ± 93
Ubatuba–SP	Captivity	UB22	53.5	99,346	2079	99	4.17 ± 0.009	2118 ± 75
Ubatuba–SP	Captivity	UB23	58.6	70,520	2330	98	5.38 ± 0.006	2802 ± 77
Ubatuba–SP	Captivity	UB24	64.7	43,980	1036	99	4.70 ± 0.001	1875 ± 34
Range			31.1–64.7	70,792–127,862	1589–2436	98–99	2.16–5.38	953–2549

Fecal samples were collected at Ubatuba and rectal samples at Praia do Forte

*CCL* curved carapace length, *BA* State of Bahia, *SP* State of Sao Paulo, *ave* average

^a^Calculated upon sample rarefaction at 43000 reads

**Table 2:** Summary statistics of general lineal models describing the relationship between indices of microbial diversity in fecal and rectal samples of wild juvenile green turtles *Chelonia mydas*, sampling area (subtropical Ubatuba and tropical Praia do Forte) and curved carapace length (CCL)

Microbial diversity		*F*	df	*p*	*r* ^2^
OTUs	Model	4.155	2.15	*0.040*	0.296
	CCL	6.016	2.16	*0.023*	
	Area	6.205	2.16	*0.028*	
Chao 1	Model	4.517	2.16	*0.032*	0.319
	CCL	6.177	2.15	*0.027*	
	Area	7.671	2.15	*0.016*	
Shannon	Model	3.180	2.16	0.075	NA
	CCL	3.939	2.15	0.069	
	Area	2.708	2.15	0.033	

Microbial diversity is higher in tropical Praia do Forte and increases with turtles size. Italics denote statistical significance

*NA* not applicable

**Fig. 1:**
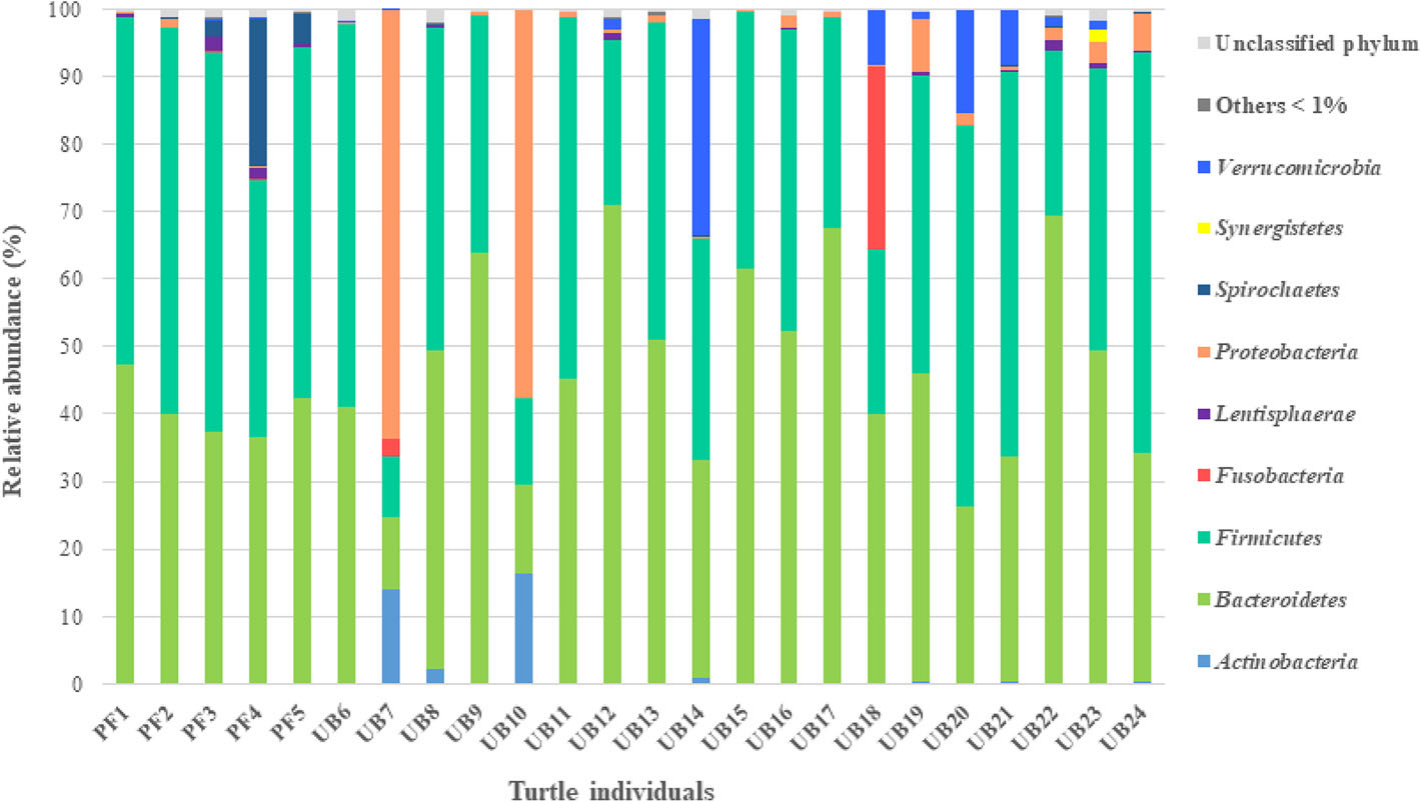
Percentages of sequences from each individual turtle, fecal or rectal sample assigned at the phylogenetic level of phylum, according to the RDP Bayesian Classifier database with a bootstrap confidence above 80%. PF1 to PF5 = wild turtles from Praia do Forte; UB6 to UB16 = wild turtles from Ubatuba; UB17 to UB24 = captive turtles from Ubatuba. Taxa with a RA lower than 1% is grouped as “others”

**Fig. 2:**
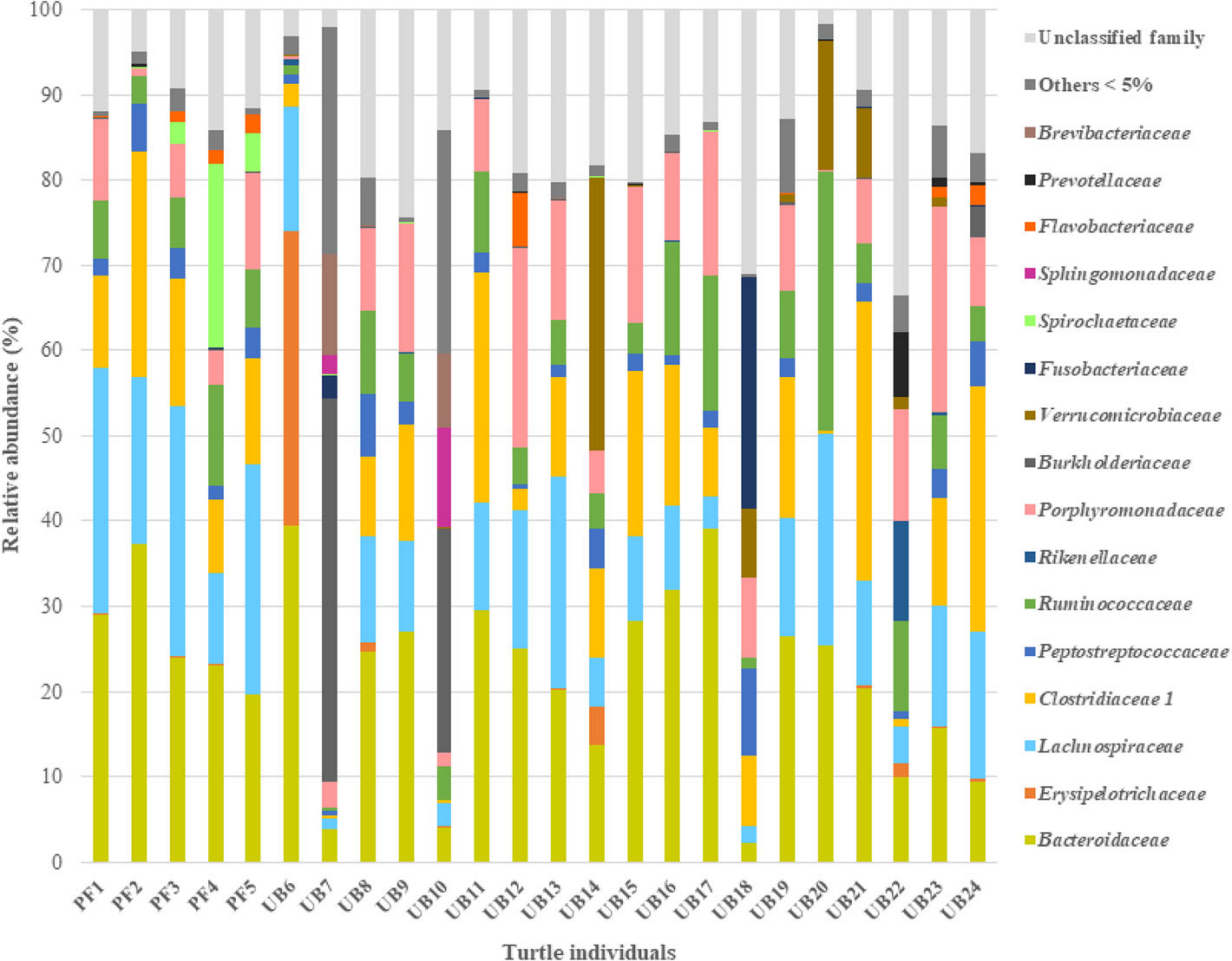
Percentages of sequences from each individual turtle, fecal or rectal sample assigned at the phylogenetic level of family, according to the RDP Bayesian Classifier database with a bootstrap confidence above 80%. PF1 to PF5 = wild turtles from Praia do Forte; UB6 to UB16 = wild turtles from Ubatuba; UB17 to UB24 = captive turtles from Ubatuba. Taxa with a RA lower than 5% is grouped as “others”

The dominant phyla in the majority of wild and captive turtles were *Bacteroidetes*, ranging 20–70% of relative abundance (RA), and *Firmicutes* with a 24–56% of RA (Fig. 1). In most of the studied turtles (Fig. 2), the predominant families within *Bacteroidetes* phylum were *Bacteroidaceae* and *Porphyromonadaceae*, while within *Firmicutes* phylum the predominant families were *Clostridiaceae*, *Lachnospiraceae*, and *Ruminococaceae*, with the exception of two wild individuals and one captive individual from Ubatuba. The bacterial community structure of these two anomalous wild turtles (UB7 and UB10) was characterized by a high RA of representatives from the phyla *Proteobacteria* (approximately 60% RA) and *Actinobacteria*, which in this latter phylum belonged to the *Mycobacterium* genus (1.2 and 4.7% RA in UB7 and UB10, respectively). The main OTUs of the former *Proteobacteria* phylum were related to *Burkholderia* spp. (*Betaproteobacteria*), *Sphingopyxis* spp. (*Alphaproteobacteria*), and *Pseudomonas* spp. (*Gammaproteobacteria*), which combined represented 49.5 and 38.3% RA for UB7 and UB10, respectively. Except for *Sphingopyxis*, these genera have been associated to the presence of *Staphylococcus* spp., in the phylum *Firmicutes* (5.0% and 3.5% of RA in UB7 and UB10, respectively). Regarding the bacterial community of the anomalous captive turtle (UB18), it was characterized by a high abundance of the phylum *Fusobacteria* (27% RA). Such dominance was primarily caused by OTU7, affiliated with the microaerotolerant fermentative *Cetobacterium* sp. (96% of similarity to *Cetobacterium ceti*), also found in whale, dolphin, and porpoise gut flora (Bik et al. 2016).

When those three anomalous turtles (UB7, UB10, and UB18) were removed from the analysis, the abundance of *Proteobacteria* was consistently higher in captive (range 0.7–7.7% RA) than in wild (range 0.2–1.9% RA) turtles from Ubatuba (Mann-Whitney test; *U* = 57.00, *p* = 0.046). On the other hand, *Akkermansia* spp., belonging to the phylum *Verrumicrobia*, was found with a RA of 8–15% in captive turtles UB18, UB20, and UB21. It is noteworthy that in one of the wild individuals (UB14), *Akkermansia* was enriched up to a 30% of RA and, curiously, the microbiome of this individual was rather different from that of other wild turtles.

The family *Clostridiaceae* comprised a ribotype (OTU1) that was predominant in almost all samples (from 1 to 8% RA). OTU1 belongs to the unclassified *Clostridiaceae* 1 subfamily. Interestingly, the RA of OTU1 in the wild turtles with the most dissimilar microbiome (UB7 and UB10) was < 0.1% RA (Figs. 1 and 2). Moreover, predominant OTUs of *Lachnospiraceae* and *Bacteriaceae* in those anomalous turtles were present at a comparatively low RA. On the other hand, representatives of the genus *Spirochaetes* were detected in all samples, but only in turtles from Praia do Forte this phylum appeared in significant amounts, especially in PF3, PF4, and PF5, where OTU6 was predominant. This OTU was distantly related (88% in sequence homology) to *Treponema brennaborense* and might therefore correspond to an undescribed species. Furthermore, samples from Praia do Forte had a lower abundance of representatives in the *Actinobacteria* and *Verrumicrobia*, when compared to the Ubatuba individuals.

**Fig. 3:**
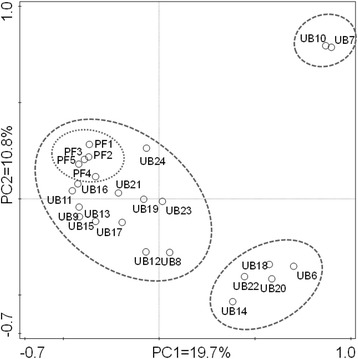
PCoA biplot of the gut microbiome in Brazilian green turtles based on the Bray-Curtis distance matrix. Wild turtles came from Praia do Forte (PF1 to PF5) and Ubatuba (UB6 to UB16). Captive turtle came only from Ubatuba (UB17 to UB24). The percentage of explained variation encompassed by the two main axes has been indicated. The main sample score clusters (dashed contours) and the more specific subcluster from Praia do Forte (dotted contour) have been highlighted

Multivariate analysis (PCoA of samples’ Bray-Curtis distances based on OTUs incidence) (Fig. 3) showed three major clusters in relation to the microbial community structure of the gut microbiome from the studied turtles (Fig. 3). The smallest and more specific group confirmed the uniqueness of the bacterial community in the two anomalous wild turtles described above, UB7 and UB10. No significant segregation was observed between wild and captive turtles, but individuals from Ubatuba displayed a significant variability, and two major groups were apparent. The minor cluster encompassed the previously described individuals that were characterized by a relatively high abundance of *Akkermansia* spp., while a second larger one also contained the samples from Praia do Forte forming a very compact subcluster.

## Discussion

Green turtles settle in the coastal habitats of the south-western South Atlantic when they are 30–45 cm in CCL [34, 41, 51]. The results reported here indicated that most of the green turtles less than 45 cm CCL from Brazil exhibited a fecal microbiota co-dominated by phyla *Bacteroidetes* and *Firmicutes* and high levels of *Clostridiaceae*, *Porphyromonas*, *Ruminococcaceae*, and *Lachnospiraceae* within the latter phylum. Furthermore, this was the only microbiota profile found in wild green turtles > 45 cm CCL and in most of the captive green turtles of any size feeding on a macroalgae/fish mixed diet. These results suggest a fast acquisition of a polysaccharide fermenting gut microbiota by juvenile green turtles after settlement into coastal habitats.

A high abundance of *Proteobacteria* had been previously reported from the cloaca of pelagic (range 17.1–21.7 cm CCL) and recently settled (29.4–34.6 cm CCL) juvenile green turtles from Florida and from the gut of omnivorous marine fishes, but not from other groups of herbivorous vertebrates (Table 3). A high abundance of *Proteobacteria* has been observed also in two wild and one captive green turtles from Brazil less than 45 cm CCL (this study), but this is probably because they were immunodepressed and not because of recent settlement. We hypothesize that the prevalence of the *Proteobacteria* phylum in those three individuals was because of lesions from anthropogenic impacts [52]. The same is true for *Mycobacterium,* from the *Actinobacteria* phylum, a genus very uncommon in turtles but which includes several well-known pathogens for reptiles and amphibians [53, 54]. Furthermore, three captive and one wild turtle shared OTUs affiliated to the *Akkermansia* genus (*Verrumicrobiaceae* family). *Akkermansia* is a mucin-degrading bacterium commonly found in the human gut and recently isolated in reptiles [55, 56]. Several studies showed that the enrichment of *Akkermansia* induces gut inflammation and is associated with colonic diseases in mammals, but nothing is known about its pathogenicity in reptiles. It is also worth noting a small captive turtle (34.9 cm CCL) with a microbiota dominated by *Bacteroidetes* and *Firmicutes* but with a high relative abundance of *Fusobacteria*, a group occurring sporadically in carnivorous marine mammals [4].

**Table 3:** Relative abundance of bacterial phyla to the gut microbiota of omnivorous and herbivorous vertebrates

Species	Diet	*Firmicutes*	*Bacteroidetes*	*Verrucomicrobia*	*Spirochaetes*	*Proteobacteria*	*Actinobacteria*	Other	Source
Teleosteans									
*Acanthurus gahhm*^1^	Omn/Alg	**29.5**	0.6	0.0	1.3	**49.4**	7.7	9.1	Miyake et al. (2015)
*Naso elegans*^1^	Herb/Alg	**97.4**	0.0	0.0	0.0	0.0	0.0	2.6	Miyake et al. (2015)
*Naso unicornis*^1^	Herb/Alg	**83.3**	9.0	**2.6**	0.0	2.6	1.3	1.2	Miyake et al. (2015)
*Siganus stellatus*^1^	Omn/Alg	**42.3**	11.5	0.0	2.6	**37.2**	0.0	6.4	Miyake et al. (2015)
Turtles									
*Chelonia mydas*^a,2^	Omn/Alg	6.5	27.1	0.6	0.0	**60.5**	0.1	5.2	Price et al. (2017)
*Chelonia mydas*^b, 2^	Herb/Seg	8.3	15.4	0.2	0.2	**66.6**	1.7	7.6	Price et al. (2017)
*Chelonia mydas*^c,e,3^	Herb/Alg	10.8	11.8	0.1	0.1	**60.7**	15.2	1.3	This study
*Chelonia mydas* ^d,3^	Herb/Alg	**44.8**	**46.6**	**3.8**	1.3	1.1	0.3	2.1	This study
*Geochelone nigra*^*3*^	Herb/Ter	**81.1**	4.4	0.1	0.0	2.0	0.8	11.6	Hong et al. (2011)
*Gopherus polyphemus*^3^	Herb/Ter	**38.4**	**36.9**	**3.0**	4.4	< 3.0	< 3.0	7.4	Yuan et al. (2015)
Lizards									
*Amblyrynchus cristatus*^3^	Herb/Alg	**75.1**	8.2	1.0	0.0	0.6	0.6	14.5	Hong et al. (2011)
*Conolophus* spp. ^3^	Herb/Ter	**63.9**	4.2	0.2	0.0	1.4	1.3	29.0	Hong et al. (2011)
*Iguana iguana*^3^	Herb/Ter	**74.0**	10.1	1.0	0.6	3.1	0.1	11.1	Hong et al. (2011)
Mammals									
*Antidorcas marsupialis*^3^	Herb/Ter	**75.6**	24.4	0.0	0.0	0.0	0.0	0.0	Ley et al. (2008)
*Dugong dugong*^3^	Herb/Seg	**57.5**	**42.5**	0.0	0.0	0.0	0.0	0.0	Eigeland et al. (2012)
*Gorilla gorilla*^3^	Herb/Ter	**67.4**	3.5	**10.5**	2.3	0.0	11.6	4.7	Ley et al. (2008)
*Loxodonta africana*^3^	Herb/Ter	**80.5**	2.5	**1.8**	0.2	10.1	4.7	0.2	Ley et al. (2008)
*Ovis canadensis*^3^	Herb/Ter	**64.0**	3.0	**2.7**	0.0	2.1	25.8	2.4	Ley et al. (2008)
*Trichechus manatus*^3^	Herb/Seg	**77.3**	19.5	0.0	0.1	0.3	2.0	0.8	Merson et al. (2014)

Bold type denote accumulated RA higher that 60%. Superscript numbers denote sample source as follows: ^1^ whole intestinal tract, ^2^ cloaca; ^3^ rectum or feces. Diet: ormnivores (Omn) or herbivores (Herb). Major group of plants in diet: algae (Alg), seagrasses (Seg) and terrestrial plants (Ter). Length of green turtles *Chelonia mydas*: ^a^ 17.1–21.7 cm CCL, ^b^ = 29.4–34.6 cm CCL, ^c^39.7–44.7, ^d^ 31.1–64.7. ^e^ potentially immunodepressed individuals

High levels of *Firmicutes* are characteristic of the gut and fecal microbiota of herbivorous vertebrates (Table 3), as this phylum plays a critical role in the fermentation of complex polysaccharides [3, 57]. The families *Ruminococcaceae* and *Lachnospiracea* are particularly relevant, as both are obligate anaerobes with capacity to degrade structural polysaccharides into short-chain volatile fatty acids [3, 58–62] and occur in large numbers only in the gut and feces of herbivorous tetrapodes [3, 8, 62, 63]. Short-chain volatile fatty acids are indeed the main product of fermentation of plant material in the large intestine of green turtles [39, 64], and the analysis of the green turtle microbiota reported here revealed that *Ruminococcaceae* and *Lachnospiraceae* represented 3–30% of the OTUs recovered from the rectal and fecal samples of most juvenile green turtles, thus confirming their capacity to ferment structural polysaccharides. This suggests that juvenile green turtles with a *Firmicutes-Bacteroidetes* dominated fecal microbiota were plant-based omnivores or herbivores, which agrees with available dietary information [31, 33, 34, 65–68].

Interestingly, *Ruminococcaceae* prevail over *Lachnospiraceae* in terrestrial herbivorous reptiles [3] but the opposite appears to be true in marine iguanas [3] and in green turtles. Macroalgae are the staple food of both groups and differ from seagrasses and terrestrial plants in high levels of sulfated polysaccharides and alginic acid and low levels of cellulose [18]. This suggests that the prevalence of *Lachnospiraceae* over *Ruminococcaceea* in marine iguanas and green turtles is related to the similar composition of the polysaccharides in their diets. Nothing is known about the microbiota of green turtles feeding on seagrasses, but the profiles of the short-chain volatile fatty acids produced in the large intestine of green turtles feeding on seagrasses and those feeding on macroalgae differ [39, 64], thus suggesting potential differences in their microbiota worth exploring in further research.

Another major difference between the rectal and fecal microbiota of green turtles and those of other herbivorous vertebrates is the high abundance of *Bacteroidetes* in the former, a pattern reported previously only from dugongs (*Dugong dugong*) and gopher tortoises (*Gopherus polyphemus*) (Table 3). *Bacteroidetes* may contribute significantly to the initial attack on both simple and complex carbohydrates [69], and Yuan et al. (2015) speculated that the high prevalence of *Bacteroidetes* in gopher tortoises might be related to the seasonally low temperatures experienced in subtropical environments. However, *Bacteroidetes* had a similar prevalence in green turtles from tropical Praia do Forte and from subtropical Ubatuba (this study), thus suggesting that seasonal differences in temperature are unlikely to not induce major changes in the relative abundance of *Bacteoidetes* and *Firmicutes*, although samples were collected in summer in both areas. A high abundance of *Bacteroidetes* is neither characteristic of the gut microbiota of herbivorous chelonians, as they represent only 4% of the relative abundance of bacteria in the microbiota of Galapagos giant tortoises (*Geochelone nigra*) [3]. It is suggested that the high presence of this phylum in all the samples of green turtles from Brazil, except those of the three anomalous individuals, could be related to the presence of high levels of organic matter in coastal waters, which allow copiotrophs (such as *Bacteroidetes*) to thrive and dominate the microbial community structure [70]. Moreover, a recent study of gut microbiota of the loggerhead sea turtle *Caretta caretta* [71] found that *Firmicutes*, *Proteobacteria*, and *Bacteroidetes* were the most predominant microbial population in turtle feces.

*Spirochaetes* is another group of non-cellulolytic bacteria associate with specific plant substrates during digestion [72], facilitating the breakdown of cellulose by co-occurring bacteria [73]. Within this phylum, the *Spirochaetes* members exhibit enormous diversity in a free-living or host-associated life, being pathogenic or non-pathogenic, and aerobic or anaerobic [74]. This phylum has also been reported to be a major component of the microbiota of gopher tortoises, omnivorous fishes and gorilla, but not in other herbivorous reptiles (Table 3). OTU 6, an unidentified *Spirochaetes*, was detected in all the samples, but only in the rectal samples of three individuals from Praia do Forte (PF3, PF4, and PF5) did it represented more than 2% of the relative abundance.

The fact that *Bacteroidetes* and *Firmicutes* were the dominant bacteria in the feces and the rectal samples of most juvenile green turtles less than 45 cm CCL, including four specimens ranging 31.1–35.0 cm CCL, indicates that they acquired a microbiota adapted to digest polysaccharides shortly after settlement. How this specialized bacterial flora is acquired by settlers remains unknown, but land and marine iguanas have been observed consuming conspecific excrements [17, 75], which certainly facilitate acquiring a plant degrading microbiota. Juvenile green turtles are not gregarious, but may form dense aggregations [31, 76], which might facilitate feces consumption and hence the quick acquisition of a bacterial flora adapted to digest polysaccharides. Alternatively, fermenters might be transferred through the diet, as they can be associated with algal surfaces [77].

Algae and seaweeds are typically rich in sulfated polysaccharides that are absent in terrestrial plants. Hence, microbiota from the phylosphere of seaweeds are characterized by high copy numbers of sulfatases in their genomes [78]. A recent study suggested that traditional sushi food, which is largely composed of seaweeds, significantly affected the gut microbiome of the Japanese population [79, 80]. It was then observed that carbohydrate-active enzymes (CAZymes) in the gut microbiome, which are absent in the human genome, were acquired by horizontal gene transfer (HGT) from the marine bacteria associated with seaweeds. Moreover, [81], reviewed several studies on the HGT phenomena between environmental and gut bacteria within the phyla of *Bacteroidetes* and *Firmicutes* in different organisms, including the grazer surgeonfish. Hence, it is well possible that the seaweed-based diet of turtles could similarly affect their gut microbiota by gene acquisition, considering that CAZymes and sulfatases are required for efficient seaweed degradation [82]. This topic merits further research taking advantage of the existing programs on captive breeding of green turtles by performing gut metagenomics analysis.

In any case, the fast acquisition after settlement in coastal areas of a microbiota adapted to ferment polysaccharides should enable green turtles to adopt an herbivorous diet soon after recruitment. This is the pattern reported from tropical areas [25, 83], but in warm temperate and subtropical regions, juvenile green turtles are best described as plant-based omnivore and only adults are primarily herbivores [19–21, 23, 33, 34, 37, 84, 85]. The results presented here indicate an increase in the taxonomic richness of the gut microbiome as turtles grow, but this is an unlikely explanation by the progressive ontogenetic dietary shift, because even small turtles had a high abundance of *Ruminococcaceae* and *Lachnospiraceae*. Consumption of animal material results into a slight and statistically significant increase in the relative abundance of *Proteobacteria*, as revealed by the differences between captive and wild healthy turtles, but the abundance of *Ruminococcaceae* and *Lachnospiraceae* remains high anyway. This suggests that omnivore is unlikely to reduce the capacity of green turtles to digest plant material.

Digestibility of plant material in green turtles increases with temperature [13] and the body temperature of juvenile green turtles inhabiting subtropical regions is close to that of water during winter months [86]. Conversely, the body temperatures of inactive adult green turtles can be 2 °C above water temperature thanks to gigantothermy [36], which explains why the digestibility of plant material by green turtles increases with body size even in tropical settings [13]. Interestingly, the apparent digestibility of plant material does not increase with body size in marine iguanas [86], because even very small individuals can rise significantly their body temperature through basking in black lava [17]. Green turtles bask regularly in the beaches of Hawaii and Galapagos [87, 88] and this behavior has been suggested to improve digestion, but beach basking has never been reported in other areas to our knowledge. If green turtles inhabiting subtropical and warm temperate regions do not bask in winter, the digestibility of plant material by small individuals can be compromised during winter, even if they support a specialized microbiota rich in *Ruminococcaceae* and *Lachnospiraceae*, which may explain the progressive dietary shift as they grow.

## Conclusions

This study revealed that juvenile green turtles from the coastal waters of Brazil had the same general microbiota profile, regardless of size and origin (wild vs. captive; subtropical Ubatuba vs. tropical Praia do Forte). This indicates a fast acquisition of a microbiota with capacity to ferment structural polysaccharides soon after settlement in the coastal waters of Brazil and that the regular consumption of animal prey does not significantly reduce the presence of *Ruminococcaceae* and *Lachnospiraceae* and, hence, does not impair the capacity to ferment structural polysaccharides. However, subtropical specimens displayed a larger variability in the gut microbial community structure, which in the most extreme cases was clearly related to poor physical condition. In summary, there is no reason for a delayed ontogenetic dietary shift after settlement, unless low winter temperature reduces their capacity to digest plant material.

## Additional file

Additional file 1: Phylogeny and incidence of gut eubacteria. (XLSX 1339 kb)

## Abbreviations

BA: State of Bahia
CCL: Curved carapace length
NGS: Next-generation sequencing
OTU: Operational taxonomic unit
SP: State of Sao Paulo

## References

[1] Sues H-D, Reisz RR (1998). The origin and early evolutionary history of amniotes. Trends Ecol Evol.

[2] Barboza PS, Bennett A, Lignot JH, Mackie RI, McWhorter TJ, Secor SM, Skovgaard N, Sundset MA, Wang T (2010). Digestive challenges for vertebrate animals: microbial diversity, cardiorespiratory coupling, and dietary specialization. Physiol Biochem Zool.

[3] Hong P-Y, Wheeker E, Cann IK, Mackie I (2011). Phylogenetic analysis of the fecal microbial community in herbivorous land and marine iguanas of the Galápagos Islands using 16S rRNA-based pyrosequencing. Int Soc Microb Ecol.

[4] Keenan SW, Engel AS, Elsey RM (2013). The alligator gut microbiome and implications for archosaur symbioses. Sci Rep.

[5] Mackie RI, Nelson DM, Wheeler E, Wikelski M, Cann IK (2008). Fermentative digestion in herbivorous lizards: bacterial population analysis in the intestinal tract of free-living land (*Conolophus pallidu*s) and marine iguanas (Amblyrhynchus cristatus) on the Galapagos archipelago. Multidiscip J Microb Ecol.

[6] Clauss M, Frey R, Kiefer B, Lechner-Doll M (2003). The maximum attainable body size of herbivorous mammals: morphophysiological constraints on foregut, and adaptations of hindgut fermenters. Oecologia.

[7] Edwards MS, Ullrey DE (1999). Effect of dietary fiber concentration on apparent digestibility and digesta passage in non human primates. II. Hindgut and foregut fermenting folivores. Zoo Biol.

[8] Miyake S, Nguci DK, Stingl U (2015). Diet strongly influences the gut microbiota of surgeonfishe. Mol Ecol.

[9] André JG, Gyuris E, Lawler IR (2005). Comparison of the diets of sympatric dugongs and green turtles on the Orman reefs, Torres Strait, Australia. Wildl Res.

[10] Castelblanco-Martinez DN, Morales-Vela B, Hernandez-Arana HA, Padilla-Saldivar J (2009). Diet of the manatees (*Trichechus manatus manatus*) in Chetumal Bay, Mexico. Lat Am J Aquat Mamm.

[11] Marsh H, Channells PW, Heinsohn GE, Morrissey J (1982). Analysis of stomach contents of dugongs from Queensland. Australian. Wildl Res.

[12] Mignucci-Giannoni AA, Beck CA (1998). The diet of the manatee *(Trichechus manatus*) in Puerto Rico. Mar Mamm Sci.

[13] Bjorndal KA (1980). Nutrition and grazing behavior of the green turtle *Chelonia mydas*. Mar Biol Res.

[14] Yamamuro M, Chirapart A (2005). Quality of the seagrass Halophila ovalis on a Thai intertidal flat as food for the dugong. J Oceanogr.

[15] Eigeland KA, Lanyon JM, Trott DJ, Ouwerkerk D, Blanshard W, Milinovich GJ, Guilino L-M, Martinez E, Merson S, Klieve AV (2012). Bacterial community structure in the hindgut of wild and captive dugongs, *Dugong dugon*. Aquat Mamm.

[16] Merson SD, Ouwerkerk D, Gulino L-M, Klieve A, Bonde RK, Burgess EA, Lanyon JM (2014). Variation in the hindgut microbial communities of the Florida manatee, *Trichechus manatus* latirostris over winter in Crystal River, Florida. FEMS Microbiol Rev.

[17] Wikelski M, Trillmich F (1993). Foraging strategies of the Galapagos marine iguana (*Amblyrhynchus cristatus*): adapting behavioral rules to ontogenetic size change. Behaviour.

[18] Graham L, Wilcox L (2000). Algae.

[19] Arthur KE, Boyle MC, Limpus CJ (2008). Ontogenetic changes in diet and habitat use in green sea turtle (*Chelonia mydas*) life history. Mar Ecol Prog Ser.

[20] Cardona L, Campos P, Levy Y, Demetropoulos A, Margaritoulis D (2010). Asynchrony between dietary and nutritional shifts during the ontogeny of green turtles (*Chelonia mydas)* in the Mediterranean. J Exp Mar Biol Ecol.

[21] Cardona L, Aguilar A, Pazos L (2009). Delayed ontogenic dietary shift and high levels of omnivory in green turtles (*Chelonia mydas*) from the NW coast of Africa. Mar Biol.

[22] Gonzalez Carman V, Falabella V, Maxwell S, Albareda D, Campagna C, Mianzan H (2012). Revisiting the ontogenetic shift paradigm: the case of juvenile green turtles in the SW Atlantic. J Exp Mar Biol Ecol.

[23] Howell LN, Reich KJ, Shaver DJ, Landry Jr AM, Gorga CC (2016). Ontogenetic shifts in diet and habitat of juvenile green sea turtles in the northwestern Gulf of Mexico. Mar Ecol Prog Ser.

[24] Parker D, Dutton PH, Balazs GH (2011). Oceanic diet and distribution of haplotypes for the green turtle, *Chelonia mydas,* in the central North Pacific. Pac Sci.

[25] Reich KJ, Bjorndal KA, Bolten AB (2007). The ‘lost years’ of green turtles: using stable isotopes to study cryptic life stages. Biol Lett.

[26] Bjorndal KA, Bjorndal KA (1995). The consequences of herbivory for the life history pattern of the Caribbean green turtle, Chelonia mydas. Biology and conservation of sea turtles revised edition.

[27] Burkholder DA, Heithaus MR, Thomson JA, Fourqurean JW (2011). Diversity in trophic interactions of green sea turtles Chelonia mydas on a relatively pristine coastal foraging ground. Mar Ecol Prog Ser.

[28] Carrión-Cortez JA, Zárate P, Seminoff JA (2010). Feeding ecology of the green sea turtle (*Chelonia mydas*) in the Galapagos Islands. J Mar Biolog Assoc UK.

[29] Ferreira MM (1968). On the feeding habits of the green turtle *Chelonia mydas* along the coast of the state of Ceara. Arquivos da Estacao de Biologia Marinha da Universidade do Ceara.

[30] Mortimer JA, Bjorndal KA (1982). Feeding ecology of sea turtles. Biology and conservation of sea turtles.

[31] Reisser J, Proietti M, Sazima I, Kinas P, Horta P, Secchi E (2013). Feeding ecology of the green turtle (*Chelonia mydas)* at rocky reefs in western South Atlantic. Mar Biol.

[32] Russell DJ, Balazs GH (2009). Dietary shifts by green turtles (*Chelonia mydas)* in the Kane’ohe bay region of the Hawaiian islands: a 28-year study. Pac Sci.

[33] Santos RG, Silva Martins A, Batista MB, Horta PA (2015). Regional and local factors determining green turtle *Chelonia mydas* foraging relationships with the environment. Mar Ecol Prog Ser.

[34] Vélez-Rubio GM, Cardona L, López-Mendilaharsu M, Martínez Souza G, Carranza A, González-Paredes D, Tomás J (2016). Ontogenetic dietary changes of green turtles (*Chelonia mydas)* in the temperate southwestern Atlantic. Mar Biol.

[35] Rey FE, Gonzalez MD, Cheng J, Wu M, Ahern PP, Gordon JI (2013). Metabolic niche of a prominent sulfate-reducing human gut bacterium. Proc Natl Acad Sci.

[36] Standora EA, Spotila JR, Foley RE (1982). Regional endothermy in the sea turtle, *Chelonia mydas*. J Therm Biol.

[37] Arthur B, Hindell M, Bester M, Trathan P, Jonsen I, Staniland I, Oosthuizen WC, Wege M, Le MA (2015). Return customers: foraging site fidelity and the effect of environmental variability in wide-ranging Antarctic fur seals. PLoS One.

[38] Bjorndal KA. Nutritional ecology of sea turtles. Copeia. 1985;1985:736–51.

[39] Bjorndal KA, Suganuma H, Bolten AB (1991). Digestive fermentation in green turtles, *Chelonia mydas*, feeding on algae. Bull Mar Sci.

[40] Price JT, Paladino FV, Lamont MM, Witherington BE, Bates ST, Soule T (2017). Characterization of the juvenile green turtle (Chelonia mydas) microbiome throughout an ontogenetic shift from pelagic to neritic habitats. PLoS One.

[41] Gallo BMG, Macedo S, Giffoni BB, Becker JH, Barata PCR (2006). Sea turtle conservation in Ubatuba, southeastern Brazil, a feeding area with incidental capture in coastal fisheries. Chelonian Conserv Biol.

[42] Moraes SS, Machado AJ (2003). Avaliação das condições hidrodinâmicas de dois recifes costeiros do Litoral Norte do estado da Bahia. Revista Brasileira de Gerociências.

[43] Silva BM, Bugoni L, Almeida BA, Giffoni BB, Alvarenga FS, Brondizio LS, Becker JH (2017). Long-term trends in abundance of green sea turtles (Chelonia mydas) assessed by non-lethal capture rates in a coastal fishery. Ecol Indic.

[44] Dowd S, Callaway T, Wolcott R, Sun Y, McKeehan T, Hagevoort R, Edrington T (2008). Evaluation of the bacterial diversity in the feces of cattle using 16s rDNA bacterial tag-encoded flx amplicon pyrosequencing. BMC Microbiol.

[45] Caporaso G, Kuczynski J, Stombaugh J, Bittinger K, Bushman FD, Costello EK, Knight R (2010). QIIME allows analysis of high-throughput community sequencing data. Nat Methods.

[46] Reeder J, Knight R (2010). Rapidly denoising pyrosequencing amplicon reads by exploiting rank-abundance distributions. Nat Methods.

[47] Edgar RC (2010). Search and clustering orders of magni- tude faster than BLAST. Bioinformatics.

[48] Caporaso JG, Kuczynski J, Stombaugh J, Bittinger K, Bushman FD, Costello EK, Fierer N, Peña AG, Goodrich JK, Gordon JI (2010). QIIME allows analysis of high-throughput community sequencing data. Nat Methods.

[49] Haas BJ, Gevers D, Earl AM, Feldgarden M, Ward DV, Giannokous G, Ciulla D, Tabaa D, Highlander SK, Sodergren E (2011). Chimeric 16S rRNA sequence formation and detection in sanger and 454–pyrosequenced PCR amplicons. Genome Res.

[50] DeSantis TZ, Hugenholtz P, Larsen N, Rojas M, Brodie EL, Keller K (2006). Greengenes, a chimera-checked 16S rRNA gene database and workbench compatible with ARB. Appl Environ Microbiol.

[51] Gonzalez Carman V, Botto F, Gaitán E, Albareda D, Campagna C, Mianzan H (2014). A jellyfish diet for the herbivorous green turtle *Chelonia mydas* in the temperate SW Atlantic. Mar Biol.

[52] Orós J, Torrent O, Calabuig P, Déniz S (2005). Diseases and causes of mortality among sea turtles stranded in the Canary Islands, Spain (1998–2001). Dis Aquat Org.

[53] Donnelly K, Waltzek TB, Wellehan JFX, Stacy NI, Chadam M, Stacy AS (2016). Mycobacterium haemophilum infection in a juvenile leatherback sea turtle *(Dermochelys coriacea)*. J Vet Diagn Investig.

[54] Rhodin AGJ, Anver MR (1977). Myobacteriosis in turtles: cutaneous and hepatosplenic involvement in a *Phrynops hilari*. J Wildl Dis.

[55] Ouwerkerk JP, Koehorst JJ, Schaap PJ, Ritari J, Paulin L, Belzer C, de Vos WM (2017). Complete genome sequence of Akkermansia glycaniphila strain PytT, a mucin-degrading specialist of the reticulated python gut. Genome Announc.

[56] Rawski M, Kierończyk B, Długosz J, Świątkiewicz S, Józefiak D (2016). Dietary probiotics affect gastrointestinal microbiota, histological structure and shell mineralization in turtles. PLoS One.

[57] Xu J, Bjursell MK, Himrod J, Deng S, Carmichael LK, Chiang HC, Hooper LV, Gordon JI (2003). A genomic view of the human–bacteroides thetaiotaomicron symbiosis. Science.

[58] Biddle A, Stewart L, Blanchard J, Leschine S (2013). Untangling the genetic basis of fibrolytic specialization by Lachnospiraceae and Ruminococcaceae in diverse gut communities. Diversity.

[59] Flint HJ, Bayer EA, Rincon MT, Lamed R, White BA (2008). Polysaccharide utilization by gut bacteria: potential for new insights from genomic analysis. Nat Rev Microbiol.

[60] Mountfort DO, Campbell J, Clements KD (2002). Hindgut fer- mentation in three species of marine herbivorous fish. Appl Environ Microbiol.

[61] Pope PB, Denman SE, Jones M, Tringe SG, Barry K, Malfatti SA (2010). Adaptation to herbivory by the Tammar wallaby includes bacterial and glycoside hydrolase profiles different from other herbivores. Proc Natl Acad Sci.

[62] Yuan ML, Dean SH, Longo AV, Rothermel BB, Tuberville TD, Zamudio KR (2015). Kinship, inbreeding and fine-scale spatial structure influence gut microbiota in a hindgut-fermenting tortoise. Mol Ecol.

[63] Meehan CJ, Beiko RG, Phylogenomic View A (2014). Of ecological specialization in the Lachnospiraceae, a family of digestive tract-associated bacteria. Genome Biol Evol.

[64] Bjorndal KA (1979). Cellulose digestion and volatile fatty acid production in the green turtle, *Chelonia mydas*. Comp Biochem Physiol.

[65] Gama LR, Domit C, Broadhurst MK, Fuentes MMPB, Millar RB (2016). Green turtle *Chelonia mydas* foraging ecology at 25°S in the western Atlantic: evidence to support a feeding model driven by intrinsic and extrinsic variability. Mar Ecol Prog Ser.

[66] Morais RA, Santos RG, Longo GO, Yoshida ETE, Stahelin GD, Horta PA (2014). Direct evidence for gradual ontogenetic dietary shift in the green turtle, Chelonia mydas. Chelonian Conserv Biol.

[67] Nagaoka S, Martins A, Santos R, Tognella M, Oliveira Filho E, Seminoff JA (2012). Diet of juvenile green turtles (*Chelonia mydas*) associating with artisanal fishing traps in a subtropical estuary in Brazil. Mar Biol.

[68] Santos RG, Martins AS, Farias JDN, Horta PA (2011). Coastal habitat degradation and green sea turtle diets in southeastern Brazil. Mar Pollut Bull.

[69] Shah HN, Gharbia SE (1993). Ecophysiology and taxonomy of Bacteroides and related taxa. Clin Infect Dis.

[70] Troussellier M, Escalas A, Bouvier T, Mouillot D. Sustaining rare marine microorganisms: macroorganisms as repositories and dispersal agents of microbial diversity. Front Microbiol. 2017;8:947. 10.3389/fmicb.2017.00947. 10.3389/fmicb.2017.00947 PMC5447324 28611749

[71] Abdelrhman KFA, Bacci G, Mancusi C, Mengoni A, Serena F, Ugolini A (2016). A first insight into the gut microbiota of the sea turtle *Caretta caretta*. Front Microbiol.

[72] Bekele AZ, Koike S, Kobayashi Y (2011). Phylogenetic diversity and dietary association of rumen Treponema revealed using group-specific 16S rRNA gene-based analysis. FEMS Microbiol Lett.

[73] Kudo H, Cheng KJ, Costerton JW (1987). Interactions between Treponema bryantii and cellulolytic bacteria in the in vitro degradation of straw cellulose. Can J Microbiol.

[74] Gupta RS (2016). Impact of genomics on the understanding of microbial evolution and classification: the importance of Darwin’s views on classification. FEMS Microbiol Rev.

[75] Troyer K (1982). Transfer of fermentative microbes between generations in a herbivorous lizard. Science.

[76] Bresette MJ, Witherington BE, Herren RM, Bagley DA, Gorham JC, Traxler SL, Crady CK, Hardy R (2010). Size-class partitioning and herding in a foraging group of green turtles Chelonia mydas. Endanger Species Res.

[77] Ibrahim HAH, Beltagy EA, El-Din NGS, Zokm GM, El-Sikaily AM, Abu-Elela GM (2015). Seaweeds agarophytes and associated epiphytic bacteria along Alexandria coastline, Egypt, with emphasis on the evaluation and extraction of agar and agarose. Rev Biol Mar Oceanogr.

[78] Wasmund K, Mußmann M, Loy M. The life sulfuric: microbial ecology of sulfur cycling in marine sediments. Environ Microbiol Rep. 2017;9:323–344. 10.1111/1758-2229.12538 PMC5573963 28419734

[79] Hehemann JH, Kelly AG, Pudlo NA, Martens EC, Boraston AB (2012). Bacteria of the human gut microbiome catabolize red seaweed glycans with carbohydrate-active enzyme updates from extrinsic microbes. Proc Natl Acad Sci.

[80] Hehemann JH, Correc G, Barbeyron T, Helbert W, Czjzek M, Michel G (2010). Transfer of carbohydrate-active enzymes from marine bacteria to Japanese gut microbiota. Nature.

[81] Thomas F, Hehemann JH, Rebuffet E, Czjzek M, Michel G. Environmental and gut bacteroidetes: the food connection. Front Microbiol. 2011;2:93. 10.3389/fmicb.2011.00093. 10.3389/fmicb.2011.00093 PMC3129010 21747801

[82] Benjdia A, Martens EC, Gordon JI, Berteau O (2011). Sulfatases and a radical S-adenosyl-L-methionine (AdoMet) enzyme are key for mucosal foraging and fitness of the prominent human gut symbiont, Bacteroides thetaiotaomicron. J Biol Chem.

[83] Stringell TB, Clerveaux WV, Godley BJ, Kent FEA, Lewis EDG, Marsh JE, Phillips Q, Richardson PB, Sanghera A, Broderick AC (2016). Taxonomic distinctness in the diet of two sympatric marine turtle species. Mar Ecol.

[84] Lemons G, Lewison R, Komoroske L, Gaos A, Lai CT, Dutton P, Eguchi T, LeRoux R, Seminoff JA (2011). Trophic ecology of green sea turtles in a highly urbanized bay: insights from stable isotopes and mixing models. J Exp Mar Biol Ecol.

[85] Williams NC, Bjorndal KA, Lamont MM, Carthy RR (2013). Winter diets of immature green turtles (*Chelonia mydas)* on a northern feeding ground: integrating stomach contents and stable isotope analysis. Estuar Coasts.

[86] Read MA, Grigg GC, Limpus CJ (1996). Body temperatures an winter feeding in immature green turtles, *Cheloni mydas*, in Moreton Bay, south east Queensland. J Herpetol.

[87] Snell H, Fritts TH (1983). The significance of diurnal terrestrial emergence of green turtles *(Chelonya mydas)* in the Galápagos archipelago. Biotropica.

[88] Whitton GC, Balaz GH (1982). Basking behavior of the Hawaiian green turtle (*Chelonia mydas)*. Pac Sci.

